# Supplementary material for: A cell cycle-independent, conditional gene inactivation strategy for differentially tagging wild-type and mutant cells
Source: eLife. 2017 May 31;6:e26420. doi: 10.7554/eLife.26420 (PMC5493436; doi:10.7554/eLife.26420)
Supplement: Figure 1—source data 1. — DOI: http://dx.doi.org/10.7554/eLife.26420.004 [file elife-26420-fig1-data1.docx]

**Figure 1-Source data 1:** List of constructs generated in this study

| **Construct short name** | **Construct name** | **Splice phase** |
| --- | --- | --- |
| pFlip-Flop-P0 | pUC57-attB-FRT14-FRT-SA-Phase0-(GGS)4-EGFP-(GGS)4-SD-{SA- Phase0-T2A-mCherry-3xSTOP-SV40pA}reverse orientation-FRT14-FRT-attB | 0 |
| pFlip-Flop-P1 | pUC57-attB-FRT14-FRT-SA-Phase1-(GGS)4-EGFP-(GGS)4-SD-{SA- Phase1-T2A-mCherry-3xSTOP-SV40pA}reverse orientation-FRT14-FRT-attB | 1 |
| pFlip-Flop-P2 | pUC57-attB-FRT14-FRT-SA-Phase2-(GGS)4-EGFP-(GGS)4-SD-{SA-Phase2-T2A-mCherry-3xSTOP-SV40pA}reverse orientation-FRT14-FRT-attB | 2 |
