# Supplementary material for: A cell cycle-independent, conditional gene inactivation strategy for differentially tagging wild-type and mutant cells
Source: eLife. 2017 May 31;6:e26420. doi: 10.7554/eLife.26420 (PMC5493436; doi:10.7554/eLife.26420)
Supplement: Figure 1—source data 2. — DOI: http://dx.doi.org/10.7554/eLife.26420.005 [file elife-26420-fig1-data2.docx]

**Figure 1-Source data 2:** List of fly strains used in the study

| **Flybase ID** | **Genotype** |
| --- | --- |
| FBst0041072 | y[1] w[*]; Mi{y[+mDint2]=MIC}eff[MI05507]/TM3, Sb[1] Ser[1] |
| FBst0053104 | y[1] w[*]; Mi{y[+mDint2]=MIC}SNF4Agamma[MI09417]/TM3, Sb[1] Ser[1] |
| FBst0058668 | y[1] w[*]; Mi{y[+mDint2]=MIC}Cdep[MI12769] |
| FBst0058593 | y[1] w[*]; Mi{y[+mDint2]=MIC}Trim9[MI12525] |
| FBst0059750 | y[1] w[*]; Mi{y[+mDint2]=MIC}Sik3[MI15336]/SM6a |
| FBst0059200 | y[1] w[*]; Mi{y[+mDint2]=MIC}Nedd8[MI13776] |
| FBst0057963 | y[1] w[*]; Mi{y[+mDint2]=MIC}Ank2[MI12604] CG32373[MI12604] |
| FBst0060962 | y[1] w[*]; Mi{y[+mDint2]=MIC}ctrip[MI14762]/TM3, Sb[1] Ser[1] |
| FBst0030992 | y[1] w[*]; Mi{y[+mDint2]=MIC}Eip63E[MI00413]/TM6B, Tb[1] |
| FBst0001929 | P{ry[+t7.2]=hsFLP}12, y[1] w[*]; sna[Sco]/CyO |
| FBst0005580 | y[d2] w[1118] P{ry[+t7.2]=ey-FLP.N}2 |
| FBst0004539 | y[1] w[*]; P{w[+mC]=UAS-FLP.D}JD1 |
| FBst0004540 | w[*]; P{w[+mC]=UAS-FLP.D}JD2 |
| FBst0000458 | P{w[+mW.hs]=GawB}elav[C155] |
| FBst0006401 | w[1118]; eff[mer4]/TM3, Sb[1] |
| FBst0003340 | Df(3R)e-R1, Ki[1]/TM3, Sb[1] Ser[1] |
| FBst0007820 | w[1118]; Df(2L)Exel8026/CyO |
| FBst0007894 | w[1118]; Df(2R)Exel7157/CyO |
| FBst0003781 | Df(2L)TW3, l(2)74i[1]/CyO |
| FBst0004501 | Df(3L)RM5-1/TM6B, Tb[1] |
| FBst0007621 | w1118; Df(3R)Exel6142, P{XP-U}Exel6142/TM6B, Tb1 |
| FBst0004514 | w[*]; Df(3L)663, e[1]/TM3, Sb[1] |
